# Coexistence of plastic and partially diffusive phases in a helium-methane compound

**DOI:** 10.1093/nsr/nwaa064

**Published:** 2020-04-22

**Authors:** Hao Gao, Cong Liu, Andreas Hermann, Richard J Needs, Chris J Pickard, Hui-Tian Wang, Dingyu Xing, Jian Sun

**Affiliations:** National Laboratory of Solid State Microstructures, School of Physics and Collaborative Innovation Center of Advanced Microstructures, Nanjing University, Nanjing 210093, China; National Laboratory of Solid State Microstructures, School of Physics and Collaborative Innovation Center of Advanced Microstructures, Nanjing University, Nanjing 210093, China; Centre for Science at Extreme Conditions and The School of Physics and Astronomy, The University of Edinburgh, Edinburgh EH9 3FD, UK; Theory of Condensed Matter Group, Cavendish Laboratory, Cambridge, UK; Department of Materials Science & Metallurgy, University of Cambridge, Cambridge CB3 0HE, UK; Advanced Institute for Materials Research, Tohoku University, Sendai 980-8577, Japan; National Laboratory of Solid State Microstructures, School of Physics and Collaborative Innovation Center of Advanced Microstructures, Nanjing University, Nanjing 210093, China; National Laboratory of Solid State Microstructures, School of Physics and Collaborative Innovation Center of Advanced Microstructures, Nanjing University, Nanjing 210093, China; National Laboratory of Solid State Microstructures, School of Physics and Collaborative Innovation Center of Advanced Microstructures, Nanjing University, Nanjing 210093, China

**Keywords:** crystal structure prediction, *ab initio* molecular dynamics, *ab initio* calculations, high pressure and high temperature, melting and phase transition, collective motion

## Abstract

Helium and methane are major components of giant icy planets and are abundant in the universe. However, helium is the most inert element in the periodic table and methane is one of the most hydrophobic molecules, thus whether they can react with each other is of fundamental importance. Here, our crystal structure searches and first-principles calculations predict that a He_3_CH_4_ compound is stable over a wide range of pressures from 55 to 155 GPa and a HeCH_4_ compound becomes stable around 105 GPa. As nice examples of pure van der Waals crystals, the insertion of helium atoms changes the original packing of pure methane molecules and also largely hinders the polymerization of methane at higher pressures. After analyzing the diffusive properties during the melting of He_3_CH_4_ at high pressure and high temperature, in addition to a plastic methane phase, we have discovered an unusual phase which exhibits coexistence of diffusive helium and plastic methane. In addition, the range of the diffusive behavior within the helium-methane phase diagram is found to be much narrower compared to that of previously predicted helium-water compounds. This may be due to the weaker van der Waals interactions between methane molecules compared to those in helium-water compounds, and that the helium-methane compound melts more easily.

## Teaser

Scientists predicted unexpected stable helium-methane compounds and novel melting behaviors, providing new insights on the interior of the giant gaseous planets such as Uranus and Neptune.

## INTRODUCTION

Hydrogen and helium are the most abundant elements in the universe and are significant constituents of the planets in our solar system [1]. Superionicity of hydrogen in ice and ammonia was discovered in early works [2–4]. In the superionic state, some atoms form a fixed sublattice while others diffuse as in a liquid. These states have attracted much interest in planetary and high-pressure science for some decades [5–19], because ionic mobility affects thermal and electrical conductivity deep inside planets, and therefore their thermal evolution and ability to sustain magnetic fields. For example, it has been reported that the body centered cubic (bcc) phase of superionic ice transforms to a face centered cubic (fcc) phase, which is also superionic [11]; the latter has reportedly been seen in recent shock wave experiments [18,19]. Sun *et al.* [14] reported a superionic state in close-packed and *P*2_1_*/c* phases of ice at higher pressures, and French *et al.* [15] constructed thermodynamic potentials for the superionic bcc and fcc ice phases and calculated the phase boundary between them.

Superionicity of hydrogen in ammonia and ammonia compounds has also been studied [10,12,13,16,20,21]. Methane is also an important component of giant planets in addition to water and ammonia [17]. Superionic states have not been found in methane, due to the polymerization and release of hydrogen that occurs in methane at high pressures and temperatures [22–25]. Such polymerization may result in ‘diamond rain’ in the icy planets [26]. In addition to the superionic states, the plastic phase in molecular crystals such as ammonia, in which protons rotate around the fixed nitrogen atoms, has been reported to emerge at certain pressure and temperature ranges [10]. The plastic phase and rotational motion in water have been studied under high pressure [27–29]. Very recently, Li *et al.* [30] found that colossal barocaloric effects in plastic crystals may have potential applications in solid-state refrigeration technologies.

While hydrogen-rich compounds have been studied extensively, this does not hold for helium-containing compounds. Traditionally helium is seen as the most inert element because of its closed-shell electronic configuration, but recently helium has been found to react under high pressure with metals [31,32] and ionic compounds [33–35]. Liu *et al*. [33] attributed the reactivity of helium with ionic compounds to the lowering of the Madelung energy between ions arising from the insertion of helium. At high pressures, helium has also been reported to form van der Waals (vdW) compounds with atoms such as neon [36] and covalent molecules such as ammonia [21], water [37–39], nitrogen [40–42], carbon dioxide [43] and arsenolite [44].

Superionicity involving helium has rarely been studied. Technically, helium is expected to diffuse as a neutral entity rather than an ionic entity, but a partially diffusive state involving helium has significant implications for thermal conductivities and viscosities. One such example is the helium-iron compound FeHe [32]. The superionic phase of FeHe occurs at pressures above 2 TPa and temperatures higher than 12000 K. Several superionic phases have recently been found in helium-water mixtures, which show novel behaviors such as simultaneous superionicity of hydrogen and helium [39]. However, the possibility of compounds in the helium-methane system under high pressures and the nature of their high-temperature behavior are still open questions. This is despite these species forming significant portions of icy planetary atmospheres and mantles, respectively. Their miscibility is then relevant for the atmosphere-mantle boundary region, which is expected to feature large compositional gradients [45,46]. We have therefore investigated helium-methane mixtures at high pressures and temperatures.

## RESULTS

In this work, searches for helium-methane compounds were performed using a machine learning accelerated crystal structure prediction method [47]. Structural optimizations, *ab initio* molecular dynamics (AIMD) simulations, enthalpies and electronic structures were calculated using the Vienna *ab initio* simulation (VASP) code [48] with projector augmented-wave potentials and the Perdew-Burke-Ernzerhof exchange-correlation functional (PBE) [49]. Further details of the calculations are provided in the Supplementary Material.

**Figure 1.**
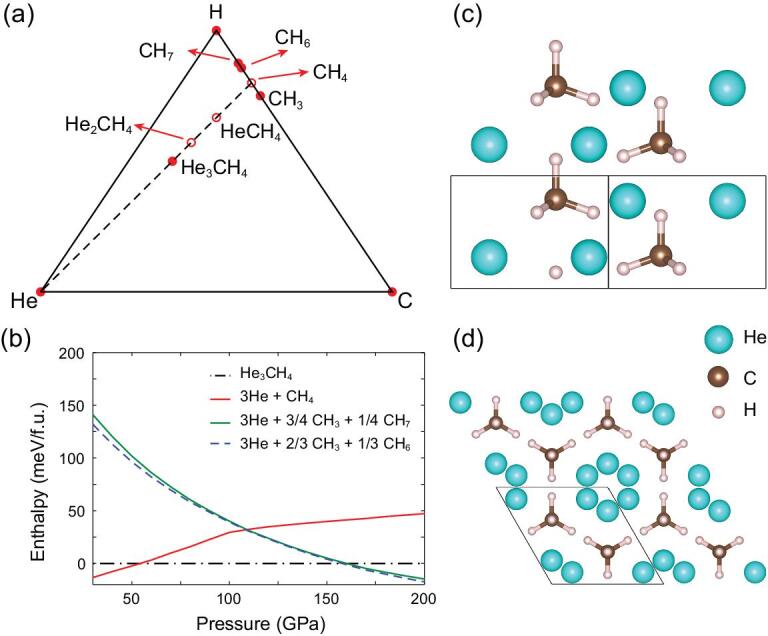
Energetic stability and crystal structures of He-CH_4_ compounds. (a) C-H-He phase diagram at 155 GPa, (b) enthalpy-pressure relations for the C-H-He compounds and the crystal structure of He_3_CH_4_ viewed along [110] (c) and [001] (d). The solid and open circles represent stable and metastable phases, respectively.

Thermodynamic stabilities of the newly predicted compounds were estimated from their relative enthalpies of formation compared with those of polymorphs of carbon, hydrogen and helium, and C—H compounds predicted in previous works [23,25,50–52]. Using an optB88-vdW functional [53,54] and hard pseudopotentials, we predicted that a new structure with a helium-methane stoichiometry of 3:1 is stable from 55 to 155 GPa (as shown in Fig. 1(a)) which is much wider than the stable pressure region of pure methane molecular crystals. Because methane decomposes above 100 GPa, here we showed a C-H-He phase diagram rather than a He-CH_4_ phase diagram. The formation energy of the compound is about 32 meV/f.u. at 110 GPa (Fig. 1(b)). We have also computed the enthalpy-pressure relations using the PBE and PBE + D3 functionals [55], as shown in the Supplementary Material, all of the results confirmed that He_3_CH_4_ is energetically stable. He_3_CH_4_ is a molecular crystal with hexagonal space group (SG) *P*6_3_*mc* composed of helium and methane molecules. As shown in Fig. 1(c) and (d), helium chains are inserted into the open channels formed by the methane molecules. The packing of methane molecules in He_3_CH_4_ is the same as in the experimental hexagonal closed packed (HCP) methane phase at low pressure [56], which is very different from the high-pressure phases of methane with orthorhombic space groups. Another compound, HeCH_4_ with SG *P*2_1_*/c*, is stable over a narrow range of pressures around 105 GPa. In addition, we have also found several metastable compounds, including HeCH_4_ with SG *P*2_1_, He_2_CH_4_ with SG *P*3_1_*m* and *P*2_1_*/m*, and He_2_(CH_4_)_3_ with SG *Cm*. These metastable phases are very close to the convex hull. The phase diagrams under different pressures are shown in the Supplementary Material.

He_3_CH_4_ is an insulator with a large band gap of 8.9 eV at 50 GPa, as shown in Fig. S11 in the Supplementary Material, it is higher than that of pure methane molecular crystals. The phonon band structures of He_3_CH_4_ at 0 and 50 GPa shown in the Supplementary Material indicate the dynamical stability of this compound. HeCH_4_ is also found to be an insulator with a large band gap and is dynamically stable. The conditions in the upper mantle regions of icy planets reach tens to hundreds of GPa and thousands of K [45]. To obtain the dynamical properties of He_3_CH_4_ we performed AIMD simulations within the pressure range 50–200 GPa and the temperature range 500–3000 K. The averaged mean squared displacements (MSD) of hydrogen, helium and carbon atoms were calculated to study phase transitions induced by temperature and pressure. According to the diffusive behaviors of the different atoms, the states of He_3_CH_4_ can be divided into four types: the solid phase, a plastic CH_4_

phase, a phase of plastic CH_4_ plus diffusive He, and the fluid phase.

**Figure 2.**
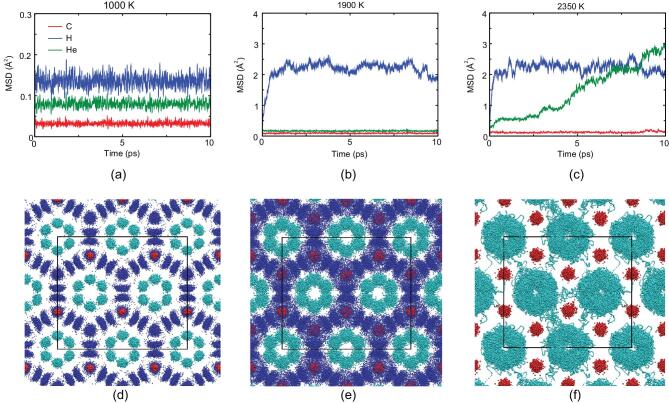
Dynamical behaviors of He_3_CH_4_ at high pressure from AIMD simulations at 1000 K, 1900 K and 2350 K. (a-c) The averaged MSD of H, He and C atoms at different temperatures. (d-f) Representations of trajectories at different temperatures in the last 10 ps. Blue, cyan and red dots represent H, He and C atoms, respectively. At 2350 K, the trajectories of H and He atoms overlap with one another, and therefore we only show He and C trajectories here.

We used three representative trajectories to reveal the differences between the states by comparing their MSD and motions, as shown in Fig. 2. All of the simulations start from the relaxed configuration at 150 GPa, which are independent, but with different temperatures. At 1000 K, the atoms are restricted to their equilibrium positions, and exhibit small vibrations. As shown in Fig. 2(a), the MSD of the atoms are extremely small in the simulations, thus He_3_CH_4_ remains in the solid phase at this temperature.

When the temperature increases to 1900 K, the compound transforms to the plastic CH_4_ phase. As shown in Fig. 2(b), the averaged MSD of H atoms increases rapidly in a short timescale, and stays about 2.0 Å^2^ afterwards. This is deemed to be a plastic phase when the methane molecules are free to rotate around the carbon atoms with small fluctuations of C-H bond lengths and angles (Fig. 2(e)). Plastic phases have also been reported in pure methane [57], ice [27,58], ammonia [10,12] and helium-ammonia compounds [21]. Meanwhile, the averaged MSD values of He and C atoms are still very small. Using the rigid molecule approximation [57] we have been able to calculate the theoretical MSD of the H atoms. Suppose that the methane molecules are rigid with fixed radius ${r_{CH}}$(the length of a C-H bond). For one of the H atoms, its initial position is ${{\boldsymbol{r}}_0}( {{r_{CH}},0,0} )$ in spherical coordinates. Since methane molecules rotate freely, the position of the H atom ${\boldsymbol{r}}( {{r_{CH}},\ \theta ,\ \phi } )$ is distributed uniformly over the sphere of radius ${r_{CH}}$. Therefore, the analytical MSD is

(1) \begin{eqnarray*}\left\langle {{{\left( {{\boldsymbol{r}} - {{\boldsymbol{r}}_0}} \right)}^2}} \right\rangle& =& \frac{1}{{4\pi }}\int_{0}^{\pi }{d}\theta \ \int_{0}^{{2\pi }}{{d\phi \sin \theta \ \left( {{\boldsymbol{r}} {-} {\boldsymbol{r}}_0^2} \right)}}\nonumber\\ &=& \frac{{2r_{CH}^2}}{{4\pi }}\int_{0}^{\pi }{d}\theta \ \int_{0}^{{2\pi }}{{d\phi \sin \theta}} \nonumber\\ &&\times\, {{(1 - \ \cos \theta )}} = \ 2r_{CH}^{2}. \end{eqnarray*}

For the plastic CH_4_ phase, the resulting MSD is$\langle {{{( {r - {r_0}} )}^2}} \rangle$ = 2.142 Å^2^, which is very close to the value from our AIMD calculations (2.169 Å^2^).

At higher temperatures (2350 K) methane molecules rotate while helium atoms are diffusive (Fig. 2(c) and (f)), which leads to the formation of a superionic-like He state in He_3_CH_4_. The diffusion coefficient of helium atoms *D_He_*is 4.81 × 10^−10^*m*^2^*/s* from the velocity auto-correlation functions (VACFs). We obtain very similar results *D_He_* = 4.48 × 10^−10^*m*^2^*/s* from the MSD. Diffusion coefficients of helium along different directions were also calculated: $D_{He}^x = \ 3.79\ \times {10^{ - 10}}{m^2}/s$, $D_{He}^y = \ 4.02\ \times {10^{ - 10}}{m^2}/s$,$\ \ D_{He}^z = \ 6.62\ \times {10^{ - 10}}{m^2}/s$. The existence of open channels along the z axis in He_3_CH_4_ (Fig. 1(d)) can explain the anisotropy of diffusion coefficients. Heating the superionic phases of He_3_CH_4_ eventually leads to melting of the methane sublattice, which gives rise to the fluid phase.

**Figure 3.**
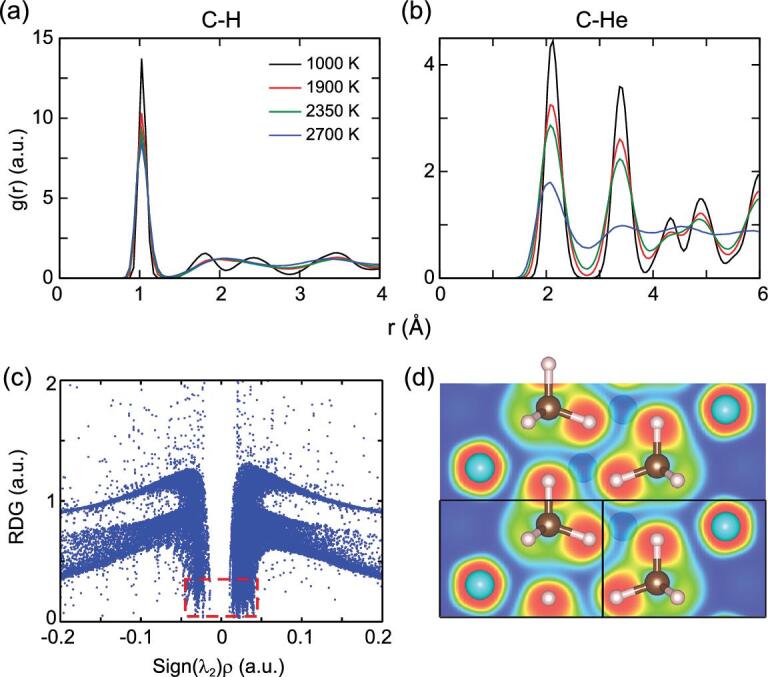
Dynamical structure and interaction analysis. Radial distribution functions (RDFs) for C-H (a) and C-He (b) pairs in He_3_CH_4_ at around 150 GPa and heating to about 1000 K (solid phase), 1900 K (plastic phase), 2350 K (coexistence of plastic and partially diffusive phase) and 2700 K (fluid phase). (c) Plots of the reduce density gradient (RDG) versus the electron density multiplied by the sign of the second largest eigenvalue of the electron-density Hessian matrix. (d) ELF plotted in the (110) plane.

We can further understand the dynamical processes of the He_3_CH_4_ structure at different temperatures by using the radial distribution function (RDF). C-H and C-He partial RDFs are shown in Fig. 3(a) and (b) and others are shown in the Supplementary Material. The C-He, He-He and H-He partial RDFs of the partially diffusive phase (2350 K, green lines) are very similar to those of the plastic phase (1900 K, red lines), but are clearly different to those of the fluid phase (2700 K, blue lines). We analyzed some trajectories of the partially diffusive phase and found that the diffusion of helium atoms maintains the order of the helium chains. In some cases of the partially diffusive phase we can see the plateaus in the averaged MSD of He. The performance of the diffusive helium atoms is similar to collective diffusion of ions in lithium battery cathode materials [59,60]. The atoms jump along the chains or hop from one chain to another and therefore the RDFs remain solid-like. However, in the fluid phase, the sublattices of helium and methane have already melted and the RDFs are liquid-like. Finally, the first peak of the C-H RDFs remains essentially unchanged deep into the fluid phase. This indicates that the integrity of the methane molecules is maintained up to the highest temperature.

The superionic state in helium-methane differs from the few other examples of superionic helium [32,39] because plastic CH_4_ and diffusive He states coexist. Plastic and superionic phases also appear in the phase diagram of ammonia and ice [10,12,58], but they cannot coexist since both the plastic and diffusive atoms in ammonia and water are protons.

**Figure 4.**
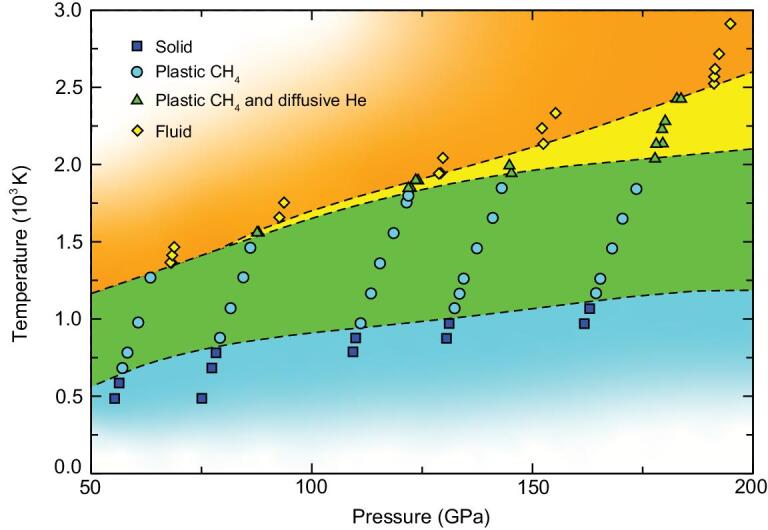
Phase diagram of He_3_CH_4_ as determined in this work. Each symbol represents an AIMD simulation. The dashed lines are phase boundaries.

From the results of extensive AIMD simulations, we proposed a phase diagram of He_3_CH_4_ between 50 and 200 GPa and below 3000 K (Fig. 4). The colored dots represent independent NVT (Canonical Ensemble) simulations which are classified by their averaged MSDs and RDFs (tests with the NPT (Isothermal-Isobaric Ensemble) confirm these classifications, see the Supplementary Material). The phase boundaries divide the diagram into four regions: solid, plastic CH_4_, plastic CH_4_ + diffusive He and fluid phases. The partially diffusive phase appears at pressures above 70 GPa within a narrow range. Compared with helium-water compounds, He_3_CH_4_ is dominated by dispersion interactions between methane molecules, which are much weaker than the hydrogen bonds between water molecules. Therefore the ice frameworks in helium-water compounds are more stable than the sublattice of methane molecules. There are many open channels in helium-water compounds, which results in a much wider partially diffusive region in helium-water than that in the helium-methane system. To validate the superionicity of helium we have performed AIMD calculations at 150 GPa using hard pseudopotentials. The results are shown in the Supplementary Material and the appearance of the partially diffusive phase at high temperatures is unaffected.

To investigate the interactions in He_3_CH_4_ we have applied a real-space analysis to the helium-methane compound. According to the electron localization function (ELF) shown in Fig. 3(d), strong intramolecular covalent bonds persist between carbon and hydrogen atoms while the interactions between methane and helium molecules are of closed-shell character. The types of intermolecular interactions in He_3_CH_4_ can be determined using the atoms-in-molecules (AIM) theory [61] and reduced density gradient (RDG) analysis [62]. Based on AIM, a topological analysis of the electron density *ρ*(**r**) was carried out to compute atomic Bader charges and search for bond critical points (BCPs). We found that the charge transfer in He_3_CH_4_ is less than 0.03*e* between carbon and hydrogen. This is consistent with the fact that the electronegativities of carbon and hydrogen are similar and methane cannot participate in hydrogen bonding. A BCP connecting a pair of atoms provides important information about the bonding between atoms. We show all of the non-equivalent BCPs and their properties in the Supplementary Material. The first and second BCPs represent the bonds inside methane molecules. The electron densities at these BCPs are much larger than others and the negative Laplacian values indicate the concentration of electrons between the carbon and hydrogen atoms. These are typical features of covalent bonding. Other BCPs with small densities and positive Laplacian values are characteristic of dispersion interactions. Furthermore, reduced density gradient analysis has been applied to the electron density. As shown in Fig. 3(c), the spikes at low density, the low-gradient region (inside the red dashed box) reflects the existence of non-covalent interactions [62] in He_3_CH_4_. The distinct spikes are very near zero, indicating that the type is vdW interactions, which are weaker than hydrogen bonds in helium-water [39] and helium-ammonia compounds [21].

## DISCUSSION

The isotopic effects in hydrogen are important in some cases [63] and we have accounted for them in the helium-methane compound by comparing the vibrational densities of states (VDOS) of He_3_CH_4_ and He_3_CD_4_ (Supplementary Material). Within the harmonic approximation, isotopic effects lead to changes in the atomic mass and in the phonon frequencies. The frequency of the highest peak in the partial VDOS drops from about 3700 cm^−1^ for hydrogen to about 2700 cm^−1^ for deuterium, with a ratio of about $1{/}\sqrt{2}$. Since strong covalent bonds exist between carbon and hydrogen/deuterium, the highest phonon peaks of carbon have the same tendencies as the isotopic effects. In contrast, the frequency region of the helium VDOS does not change substantially, because the vdW interactions between helium and methane are weak. The isotopic effects can also influence dynamical properties, and therefore we conducted AIMD calculations for He_3_CD_4_ at 2350 K and compared the VDOS of He_3_CH_4_ and He_3_CD_4_ at high temperatures. For carbon and hydrogen/deuterium the reduction of the frequencies also appears with the ratio of $1{/}\sqrt{2}$. For helium, the partial VDOS is very similar. This demonstrates that isotopic effects do not substantially affect the superionicity of the helium-methane compound.

In previous studies nuclear quantum effects were considered for ice [15] and mixtures of methane, ammonia and water [64]. The nuclear quantum corrections for He_3_CH_4_ at different densities and temperatures are shown in the Supplementary Material. The quantum corrections for the helium-methane compounds are slightly larger than those for ice [15] because of the higher proportion of hydrogen and helium in the compound and the lower temperature region in our simulations.

## CONCLUSION

In summary, we have predicted a helium-methane compound (He_3_CH_4_) that is stable at pressures relevant to upper mantle conditions of icy planets. The inclusion of He atoms highly changes the packing of methane molecules and the stable pressure region of the helium-methane compound is much wider than that of pure methane. He insertion also changes the electronic properties of methane. For example, the band gap of He_3_CH_4_ is larger than that of pure methane. Moreover, the phase diagram of He_3_CH_4_ has been investigated and a novel phase of coexistence of plastic methane and diffusive helium has been found. The temperature range of the partially diffusive regime in helium-methane is narrower than that in the helium-water system, due to the weaker interactions between the methane molecules, which results in a relatively fragile framework and an easier transition to the fluid state than in the helium-water system. In addition, we observed anisotropy in the He diffusion, which is related to the structure of the compound. We have also analyzed the interactions in He_3_CH_4_ and their effects on the phase diagram in comparison with previously predicted helium-water compounds. This work should be helpful in constructing models of icy giant planets, and it would be very instructive to investigate how much the finite-temperature miscibility of helium and methane reflects our ground state results [65].

## METHODS

We used a Bayesian-Optimization-based crystal structure prediction method [47] combined with VASP to predict new compounds for the He-CH_4_ system. The prediction results have been checked by *ab initio* random structure searching (AIRSS) [66]. The optB88-vdW functional [53] was employed to account for vdW interactions in the calculations of enthalpies and electronic structures. A basis set energy cutoff of 720 eV was used except for the AIMD calculations, for which a lower cutoff energy of 625 eV was used for reducing the computational cost of extensive AIMD simulations. The Brillouin zone was sampled with a Monkhorst-Pack k-point mesh with a spacing of 2*π* × 0.03 Å^−1^. The phonon dispersion curves were calculated using $2\ \times \ 2\ \times \ 2$ supercells with PHONOPY code [67] to validate the dynamical stabilities of the predicted structures. The AIM [61] and RDG [62] analysis of the electron density *ρ*(*r*) was performed using the critic2 code [68]. We used orthorhombic supercells containing 256 atoms to perform AIMD simulations for He_3_CH_4_ in the NVT ensemble with Γ-centered k-points sampling. The time step of AIMD was set to 1 fs and all simulations were carried out with at least 3000 steps. Some trajectories were extended to more than 10 ps to confirm the stabilities. In addition, to validate our results we employed hard pseudopotentials for hydrogen and carbon and repeated the calculations. The cutoff energy of 1000 eV was used to calculate pressure-composition phase diagrams. A cutoff energy of 910 eV was used for the AIMD simulations. The nuclear quantum corrections of free energies are calculated from the AIMD trajectories using the method proposed in reference [15].

## Supplementary Material

nwaa064_Supplemental_File
