# Supplementary material for: Coexistence of plastic and partially diffusive phases in a helium-methane compound
Source: Natl Sci Rev. 2020 Apr 22;7(10):1540–7. doi: 10.1093/nsr/nwaa064 (PMC8288639; doi:10.1093/nsr/nwaa064)
Supplement: nwaa064_Supplemental_File [file nwaa064_supplemental_file.docx]

**Supplemental Information for “Coexistence of plastic and partially diffusive phases in a helium-methane compound”**

Hao Gao,^1^ Cong Liu,^1^ Andreas Hermann,^2^ Richard J. Needs,^3^ Chris J. Pickard,^4,5^ Hui-Tian Wang,^1^ Dingyu Xing,^1^ and Jian Sun^1,*^

*1 National Laboratory of Solid State Microstructures,School of Physics and Collaborative Innovation Center of Advanced Microstructures, Nanjing University, Nanjing 210093, China*

*2 Centre for Science at Extreme Conditions and The School of Physics and Astronomy,The University of Edinburgh, Peter Guthrie Tait Road, Edinburgh EH9 3FD, United Kingdom*

*3 Theory of Condensed Matter Group, Cavendish Laboratory,J J Thomson Avenue, Cambridge CB3 0HE, UK*

*4 Department of Materials Science & Metallurgy, University of Cambridge, 27 Charles Babbage Road, Cambridge CB3 0FS, UK*

*5 Advanced Institute for Materials Research, Tohoku University 2-1-1 Katahira, Aoba, Sendai, 980-8577, Japan*

**Supplementary Figures**


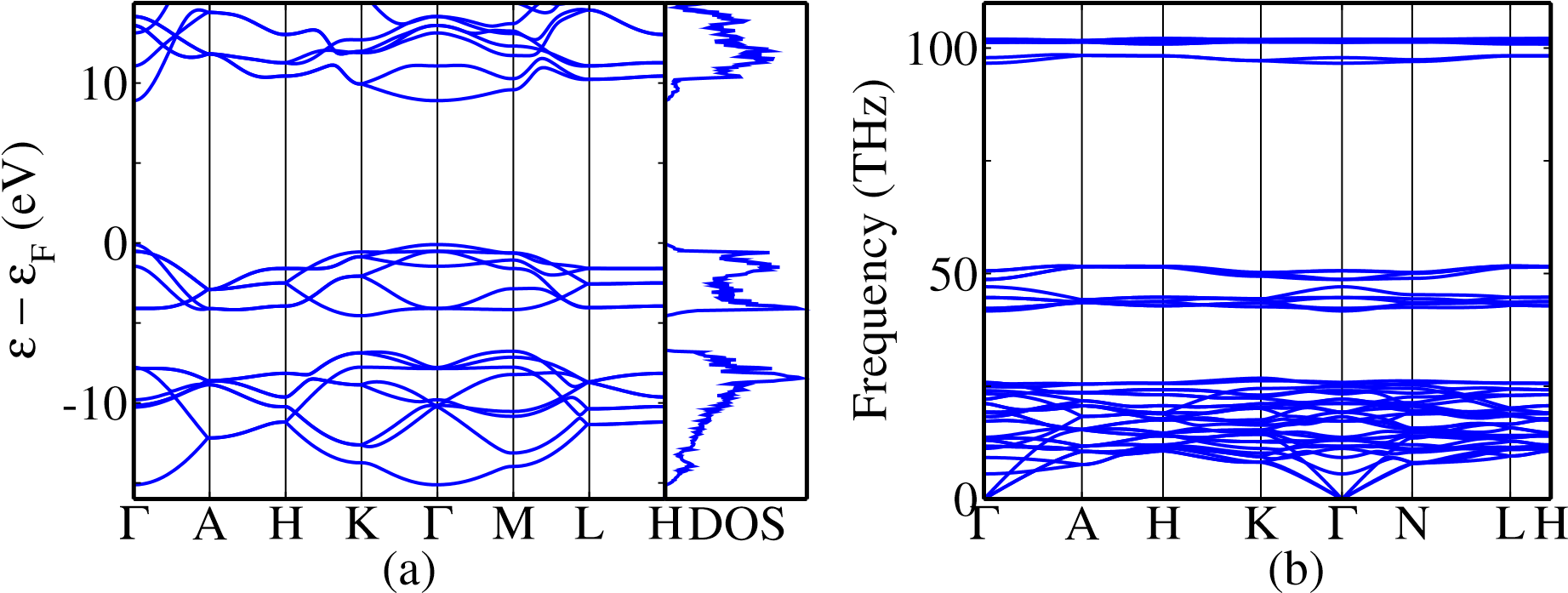


**FIG. S1.** Electronic structures(a) and phonon dispersions(b) of He_3_CH_4_ at 50 GPa.


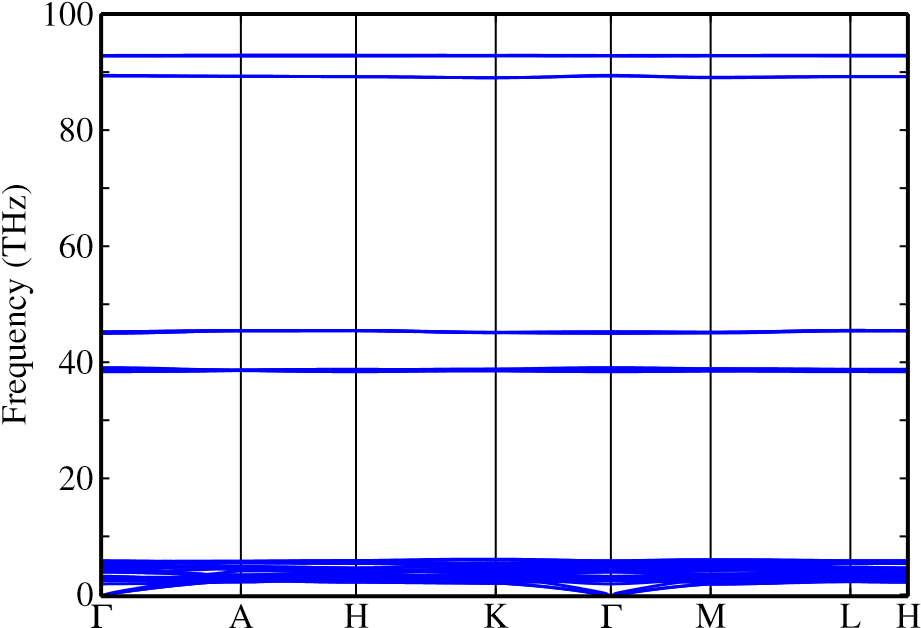


**FIG. S2.** Phonon dispersions of He_3_CH_4_ at 0 GPa.


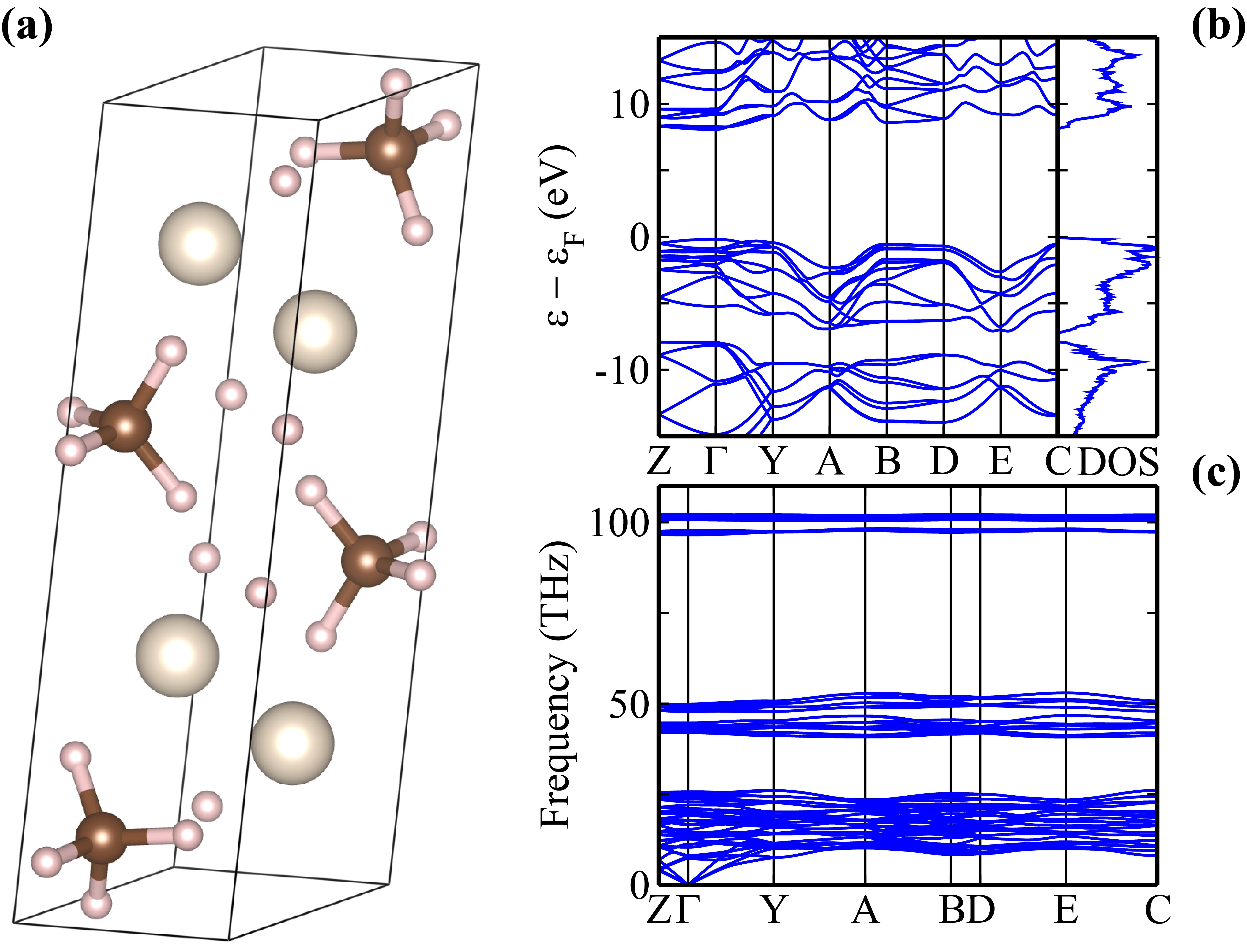


**FIG. S3.** Crystal strucuture(a), band structures(b) and phonon dispersions(c) of HeCH_4_ (*P*2_1_*/c*) at

100 GPa.


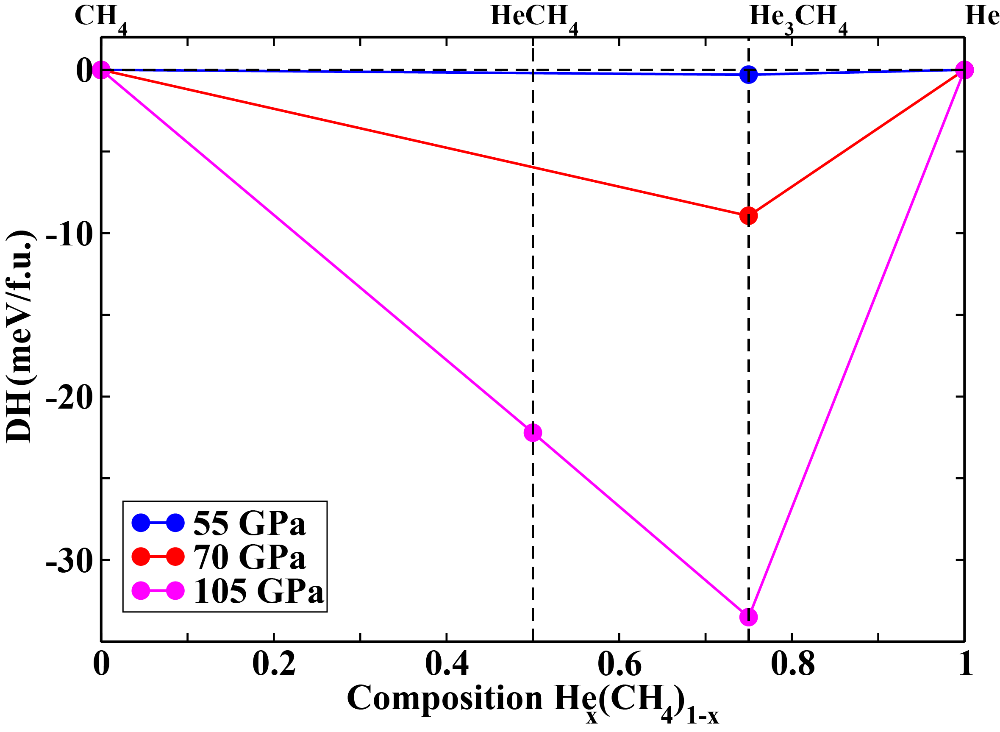


**FIG. S4.** Stability of helium-methane compounds under high pressures. Solid circles represent stable compositions. ∆*H* here is relative to the formula of He_3_CH_4_.


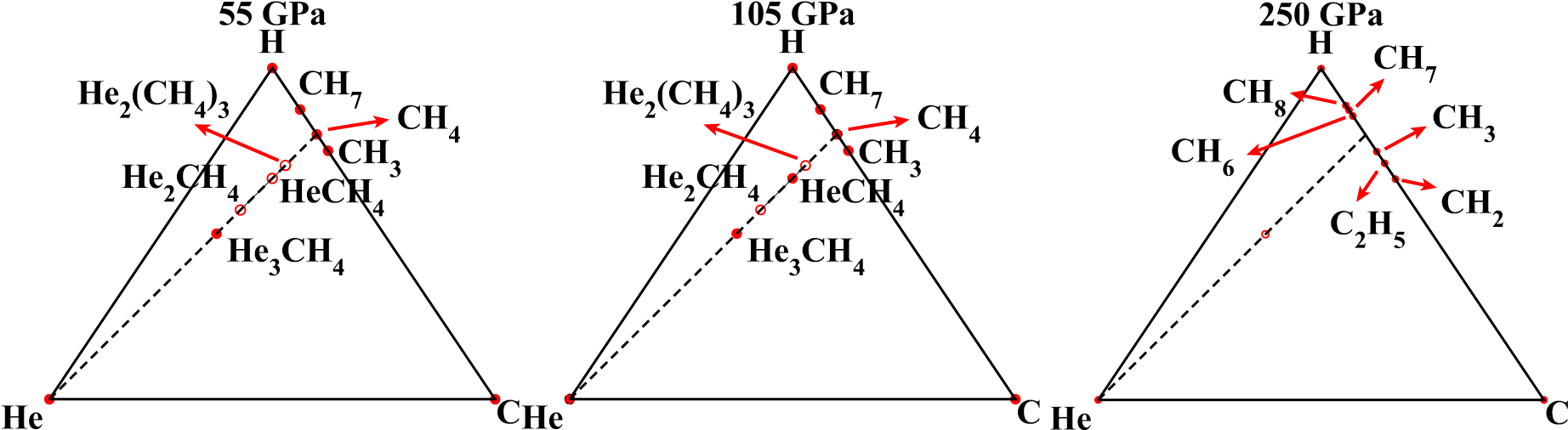


**FIG. S5.** Pressure-composition phase diagrams of C-H-He system calculated by hard pseudopotentials at different pressures. The solid and blank circles represent stable and unstable phases.


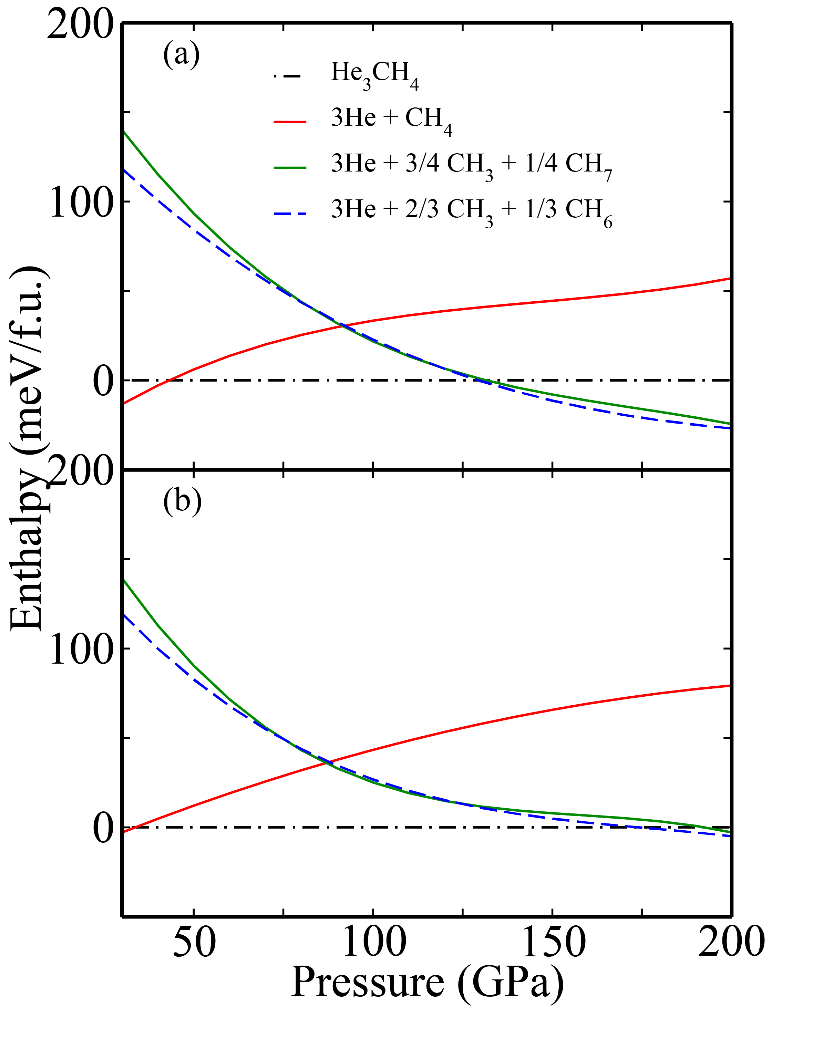


**FIG. S6.** Enthalpy-pressure relations for the C-H-He compounds calculated by PBE (a) and D3 (b)


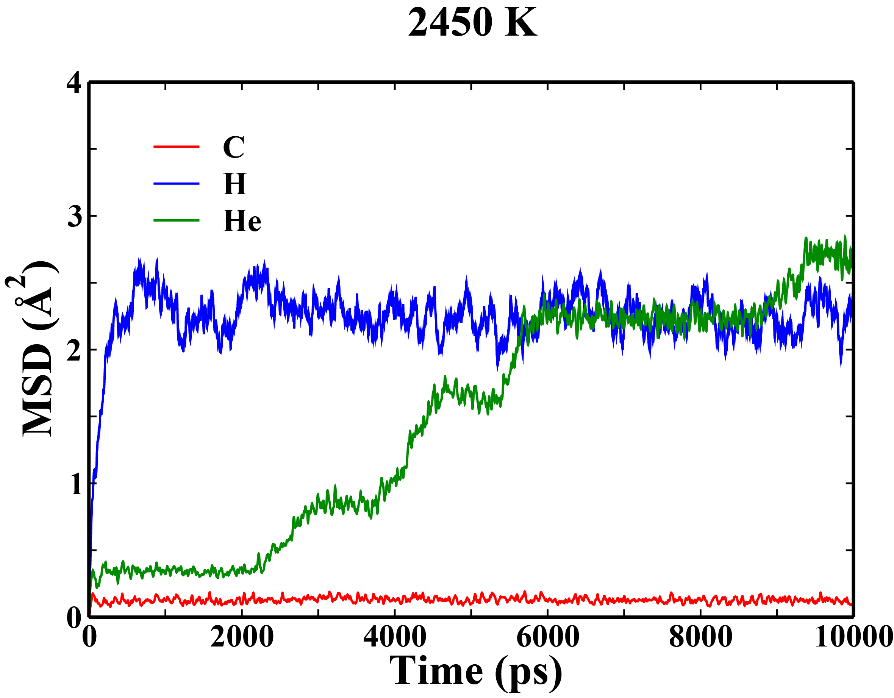


**FIG. S7.** MSD of He_3_CH_4_ at 2450 K calculated by hard pseudopotentials


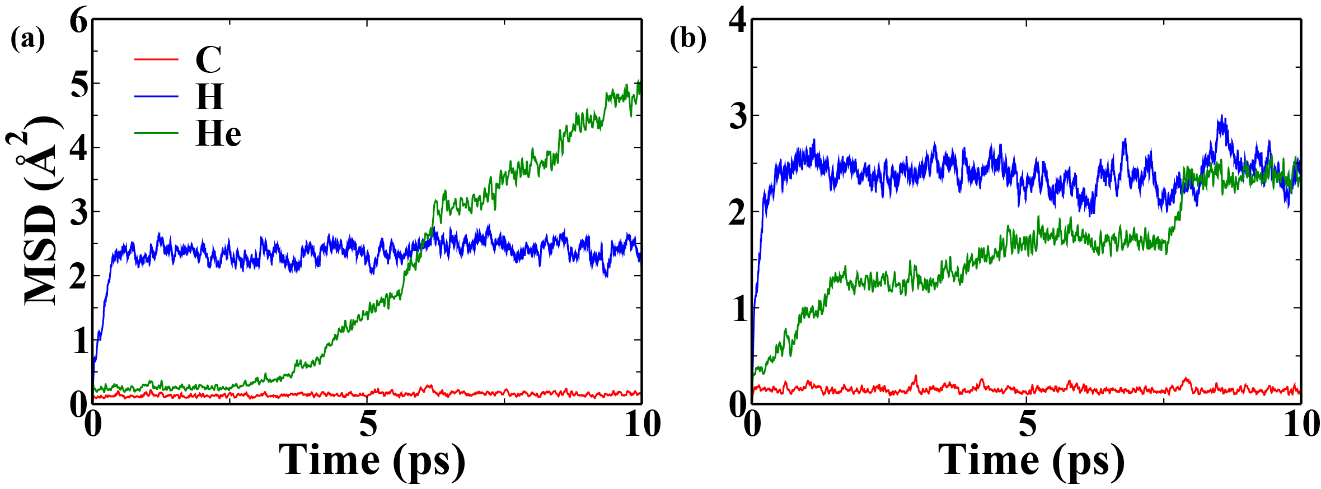


**FIG. S8.** MSD of He_3_CH_4_ at (a) 1900 K and 120 GPa, (b) 2400 K and 180 GPa in NPT ensemble


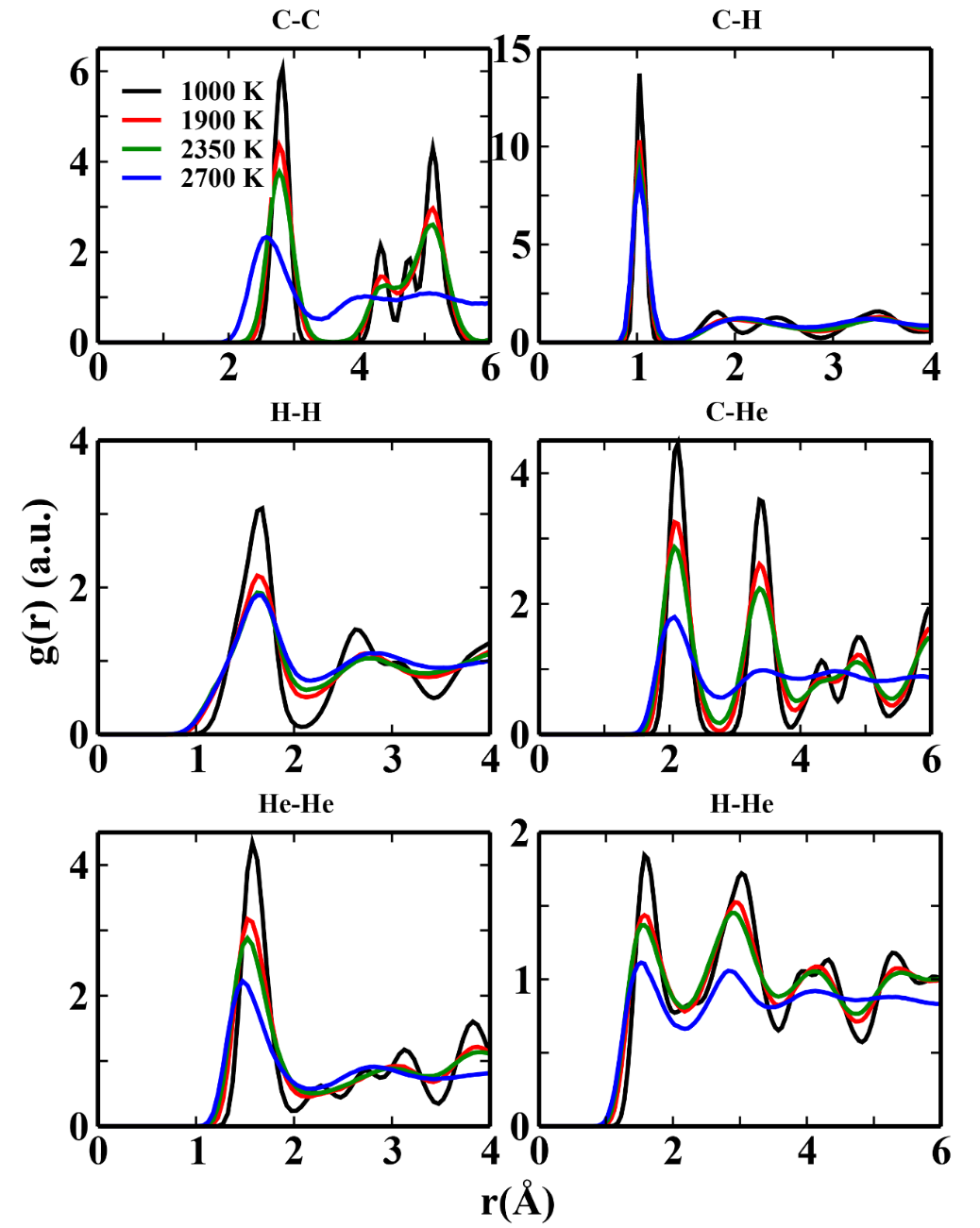


**FIG. S9.** Radial distribution functions for different pairs in He_3_CH_4_ at around 150 GPa and heating to about 1000 K (solid phase), 1900 K (plastic phase), 2350 K (coexistence of plastic and partially diffusive phase) and 2700 K (fluid phase).


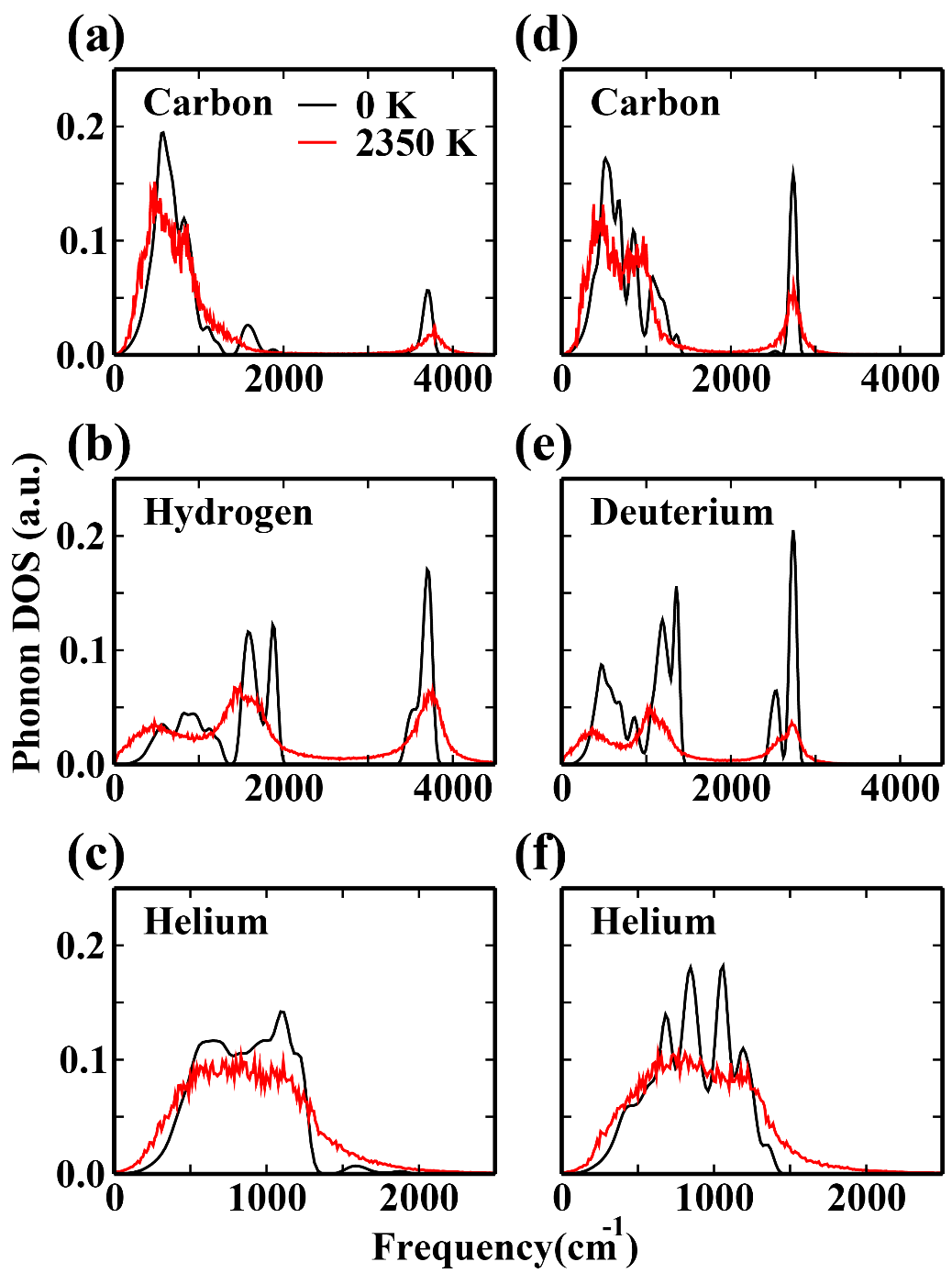


**FIG. S10.** Vibrational density of states of He_3_CH_4_ (a-c) and He_3_CD_4_ (d-f) within the harmonic approximation (0 K) and AIMD (2350 K).


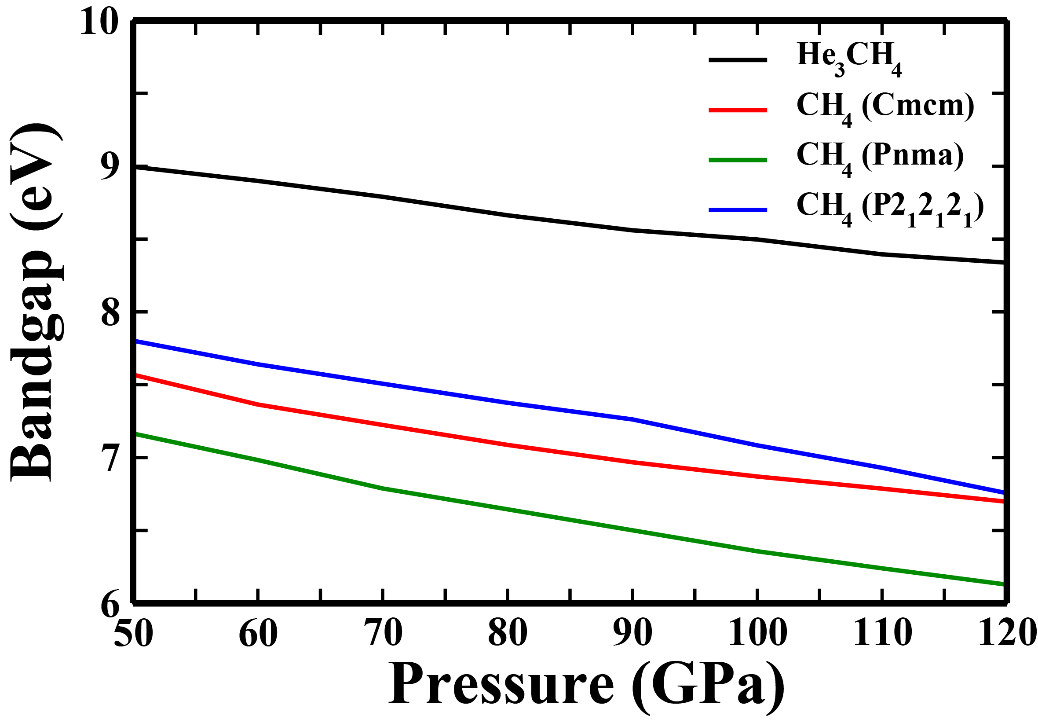


**FIG. S11.** Bandgap vs. pressure for He_3_CH_4_ and CH_4_ molecular crystals.

**Supplemental Tables**

**TABLE I**: Structure parameters of He-CH_4_ compounds

| Compound Space Group (SG No.) | | Cell Parameters | Wyckoff Positions |
| --- | --- | --- | --- |
|  | No. of formula | (Å, °) |  |
| He_3_CH_4_ | *P*6_3_*mc* (186) | a = b = 4.883, c = 3.034 | C (2b) 0.33333 0.66667 0.29147 |
|  | Z=2 | *α* = *β* = 90^◦^, *γ* = 120^◦^ | H (2b) 0.33333 0.66667 0.64216  H (6c) 0.09633 0.54816 0.17407  He (6c) 0.12194 0.24389 0.77529 |
| HeCH_4_ | *P*2_1_*/c* (14) | a = 3.100, b = 3.160, c = 9.622 | H (4e) -0.30240 -0.65741 0.50658 |
|  | Z=4 | *α* = *γ* = 90^◦^, *β* = 107.716^◦^ | H (4e) 0.24690 -0.79837 0.59726  H (4e) -0.09821 -0.63190 0.69861  H (4e) -0.19000 -1.12164 0.60739  He (4e) 0.44754 -0.19780 1.19825  C (4e) -0.08967 -0.80056 0.60368 |
| HeCH_4_ | *P*2_1_ (4) | a = 3.163, b = 3.096, c = 4.883 | C (2a) -0.09166 0.62509 0.70810 |
|  | Z=2 | *α* = *γ* = 90^◦^, *β* = 108.023^◦^ | H (2a) 0.87580 -0.03294 0.71563  H (2a) 0.22871 0.55451 0.69083  H (2a) 0.12184 0.98386 1.09993  H (2a) 0.33737 0.00147 0.47795  He (2a) 0.52492 0.59362 1.10600 |
| He_2_CH_4_ | *P*31*m* (157) | a = b = 3.161, c = 3.126 | C (1a) 0.00000 0.00000 0.83138 |
|  | Z=1 | *α* = *β* = 90^◦^, *γ* = 120^◦^ | H (1a) 0.00000 0.00000 0.49195  H (3c) 0.67988 0.00000 0.93698  He (2b) 0.33333 0.66667 0.38704 |
| He_2_CH_4_ | *P*2_1_*/m* (11) | a = 3.213, b = 5.445, c = 3.096 | H (4f) -0.10442 -0.41170 0.31155 |
|  | Z=2 | *α* = *γ* = 90^◦^, *β* = 90.178^◦^ | H (2e) 0.02612 -0.25000 -0.14948  H (2e) 0.36045 -0.25000 0.28577  He (4f) 0.46440 -0.58515 0.25003  C (2e) 0.04193 -0.25000 0.19359 |
| He_2_(CH_4_)_3_ | *Cm* (8) | a = 3.159, b = 12.845, c = 3.103 | H (4b) -0.48858 0.06910 0.58288 |
|  | Z=2 | *α* = *γ* = 90^◦^, *β* = 89.429^◦^ | H (4b) 0.73430 -0.14862 0.18022  H (4b) 1.07020 -0.14238 -0.25876  H (4b) 1.18908 -0.07414 0.20063  H (4b) 1.23070 -0.21151 0.19003  H (2a) -0.01659 0.00000 0.61433  H (2a) -0.36656 -0.00000 1.04462  He (4b) 0.56457 -0.21337 -0.36062  C (4b) 1.05838 -0.14516 0.08354  C (2a) -0.34374 0.00000 0.70131 |

**TABLE II.** Topological properties of the non-equivalent BCPs

| Atom1 | Atom2 | *ρBCP* | (*e*^−^Bohr^−3^) | ∇2*ρBCP* (*e*−Bohr−5) |
| --- | --- | --- | --- | --- |
| C | H |  | 0.307 | -1.346 |
| H | C |  | 0.306 | -1.377 |
| C | H |  | 0.030 | 0.093 |
| H | H |  | 0.027 | 0.092 |
| He | He |  | 0.023 | 0.137 |
| He | H |  | 0.027 | 0.105 |
| He | He |  | 0.021 | 0.126 |
| He | H |  | 0.025 | 0.095 |
| He | C |  | 0.019 | 0.099 |

**TABLE III.** Quantum correction of He_3_CH_4_ at different temperatures and pressures

| *ρ* (g/cm^−3^) | T (K) | P (GPa) | State | Q*_corr_* (kJ/g) |
| --- | --- | --- | --- | --- |
| 1.49 | 600 | 56 | solid | 3.33 |
| 1.49 | 1300 | 63 | plastic | 2.14 |
| 1.49 | 1500 | 68 | diffusive | 1.99 |
| 1.65 | 700 | 77 | solid | 3.00 |
| 1.65 | 1300 | 84 | plastic | 1.99 |
| 1.65 | 1600 | 88 | partially diffusive | 1.74 |
| 1.65 | 1800 | 94 | diffusive | 1.84 |
| 1.85 | 900 | 110 | solid | 2.80 |
| 1.85 | 1400 | 115 | plastic | 2.08 |
| 1.85 | 1900 | 122 | partially diffusive | 1.58 |
| 1.85 | 2100 | 130 | diffusive | 1.47 |
| 1.97 | 1000 | 131 | solid | 3.13 |
| 1.97 | 1500 | 137 | plastic | 2.04 |
| 1.97 | 2050 | 145 | partially diffusive | 1.54 |
| 1.97 | 2200 | 152 | diffusive | 1.46 |
| 2.12 | 1000 | 162 | solid | 2.87 |
| 2.12 | 1500 | 168 | plastic | 2.15 |
| 2.12 | 2350 | 180 | partially diffusive | 1.45 |
| 2.12 | 2500 | 183 | partially diffusive | 1.37 |
| 2.12 | 2700 | 191 | diffusive | 1.27 |
